# Δ4-p63 Enhances Epithelial Stem Cell Clonogenicity and Cooperates with Oncogenic KRAS to Promote Squamous Dysplasia

**DOI:** 10.21203/rs.3.rs-11038772/v1

**Published:** 2026-10-09

**Authors:** Keshia Pitt, Filipa Pinto, Thabet AlHousami, Yoshiyuki Mochida, Makoto Senoo

**Affiliations:** 1 Graduate Program in Molecular and Translational Medicine, Boston University Chobanian & Avedisian School of Medicine, 72 East Concord Street, Boston, MA 02118, USA; 2 Department of Molecular and Cell Biology, Boston University Henry M. Goldman School of Dental Medicine, 72 East Concord Street, Boston, MA 02118, USA; 3 Present Address: Wonderlab Bio LLC, 27 Drydock Ave., 2nd FL, Boston, MA 02210, USA; 4 Department of Basic & Clinical Oral Sciences, Faculty of Dentistry, Umm Al-Qura University, Makkah, Saudi Arabia; 5 Present Address: The Research Institute, Irvine, CA 92618, USA

**Keywords:** Clonogenic Culture, Oncogene, Pre-Cancer State, p63, Stem Cell, SCC

## Abstract

p63 is a critical transcription factor for the development and maintenance of stratified epithelia and is essential for the proliferative capacity of epithelial stem cells. Here, we investigated the biological significance of a Δ4-p63 isoform generated by deletion of exon 4 of the *p63* gene. Although Δ4-p63 is undetectable in normal epithelia, its expression is positively associated with human squamous cell carcinoma (SCC). Mice heterozygous for the p63 exon 4 deletion developed normally, whereas homozygous mutants were neonatal lethal. Although no overt histological abnormalities were detected in the skin of heterozygous mice, epithelial cells isolated from these animals exhibited markedly enhanced clonogenic potential compared with cells from wild-type (WT) mice. This phenotype persisted during prolonged culture and serial passage, suggesting an intrinsic increase in epithelial stem cell proliferative or self-renewal capacity rather than transient expansion associated with differentiation. Furthermore, heterozygosity for the p63 exon 4 deletion markedly enhanced epithelial dysplasia in mice expressing oncogenic Kras^G12D^, revealing a genetic interaction between p63 exon 4 deficiency and oncogenic KRAS signaling. ChIP-seq analysis demonstrated that p63 in WT mice and the p63/Δ4-p63 complex in heterozygous mice retain highly similar genome-wide DNA-binding profiles, indicating that exon 4 deletion does not abolish p63 chromatin occupancy. However, p63 and p63/Δ4-p63 showed preferential association with distinct subsets of regulatory regions. p63 in WT mice preferentially occupied regions associated with epithelial transcriptional regulators, whereas p63/Δ4-p63 in heterozygous mice preferentially associated with regulatory regions linked to Klf14, Elk4, Npas/Bmal1, Arnt, and Ar. Together, these findings suggest that Δ4-p63 is not simply a loss-of-function isoform, but instead alters the p63-dependent transcriptional landscape and epithelial stem cell state, creating a cellular context that cooperates with oncogenic KRAS to promote squamous epithelial dysplasia.

## Introduction

Stratified epithelia constitute the primary protective barrier of the body and possess a remarkable capacity for continuous self-renewal and regeneration. This capacity is sustained by epithelial stem and progenitor cells, whose proliferative potential must be precisely balanced with differentiation to maintain tissue homeostasis. Disruption of this balance can create a cellular environment permissive for malignant transformation and is a fundamental feature of squamous cell carcinogenesis [1–4].

The transcription factor p63, a member of the p53 family, is a central regulator of stratified epithelial development and homeostasis. p63 was originally identified as a p53-related gene encoding multiple protein isoforms with distinct transcriptional properties [5–8]. Genetic studies subsequently demonstrated that p63 is essential for limb, craniofacial, epidermal, and other ectoderm-derived structures [9,10]. p63-deficient mice exhibit profound abnormalities in epithelial development and fail to establish normal stratified epithelia, establishing p63 as a fundamental regulator of epithelial morphogenesis [9,10]. Subsequent studies further demonstrated that p63 is required for the proliferative capacity and long-term maintenance of stratified epithelial stem cells [11,12].

The relationship between p63 and epithelial stem cells is particularly evident in the clonogenic behavior of keratinocytes. The clonogenic assay established by Green and colleagues demonstrated that cultured keratinocytes can be classified into holoclones, meroclones, and paraclones according to their proliferative and self-renewal potential [13,14]. Highly proliferative holoclones exhibit extensive long-term proliferative capacity, whereas meroclones and paraclones progressively lose this potential as differentiation proceeds [13,14]. p63 expression is strongly associated with the highly clonogenic epithelial cell population, and experimental depletion of p63 results in loss of proliferative potential [11,12,15]. Thus, p63 is not simply a marker of epithelial stem cells but is a critical regulator of their biological properties.

The biological functions of p63 are complicated by extensive isoform diversity generated through alternative promoter usage and alternative splicing [5–8,16–19]. The two major classes of p63 proteins are generated from distinct promoters, producing TA and ΔN isoforms, while additional alternative splicing generates multiple C-terminal variants [16–19]. Among these isoforms, ΔNp63 is particularly abundant in basal keratinocytes and has important functions in epithelial development, proliferation, differentiation, and maintenance of epithelial lineage identity [20–23]. p63 interacts with lineage-specific transcription factors and chromatin regulatory machinery to establish and maintain epithelial enhancer landscapes [24]. These observations indicate that the biological activity of p63 depends not only on its expression level but also on the specific isoform expressed and the regulatory context in which it functions.

Altered p63 expression is also a prominent feature of squamous cell carcinoma (SCC). p63 is frequently expressed at high levels in SCCs and has been implicated in both the establishment and maintenance of the squamous epithelial phenotype [25–27]. Experimental studies have demonstrated that p63 can function as a critical oncogenic regulator in SCC and can cooperate with signaling pathways such as FGFR and other growth-promoting pathways to sustain tumor cell proliferation and survival [27–29]. At the same time, the precise biological consequences of p63 expression are highly dependent on isoform composition, cellular context, and the differentiation state of the target cell [16–19,25].

Our interest in the relationship between p63 isoforms and squamous carcinogenesis originated from our earlier observation that the *TP63* locus undergoes marked transcriptional dysregulation in human SCC. In that study, we identified ΔNp73L (a ΔNp63 isoform lacking exon 4) as a previously unrecognized isoform that was preferentially expressed in squamous carcinoma cells but was not detected in normal skin. Analysis of human skin cancers demonstrated frequent expression of ΔNp73L in tumors, together with loss of expression of the potential tumor suppressor p51 (TAp63r isoform) [30]. Functional studies further suggested that ΔNp73L could interfere with p53- and p51-dependent transcriptional activity [30]. These observations provided an early indication that qualitative changes in the expression of normally minor p63-family isoforms may contribute to squamous carcinogenesis.

However, the biological significance of individual alternatively spliced p63 isoforms in epithelial stem cell biology and carcinogenesis remains incompletely understood. In particular, it remains unclear whether deletion of a specific p63 exon simply reduces canonical p63 function or instead generates a qualitatively distinct p63-dependent cellular state. This distinction is potentially important because p63 regulates epithelial transcription through extensive interactions with chromatin, enhancers, and other transcription factors [24,31]. Changes in isoform structure could therefore alter not only the magnitude of p63 activity but also its genomic target selection and transcriptional output.

In the present study, we focused on a p63 isoform lacking exon 4 (or ΔNp73L), hereafter referred to as Δ4-p63. Δ4-p63 is absent in normal epithelia, whereas its expression is associated with human SCC [30]. The appearance or increased representation of an otherwise minor p63 isoform during squamous carcinogenesis raises the possibility that Δ4-p63 is not merely a consequence of aberrant splicing but may actively alter epithelial cell behavior.

This possibility is particularly intriguing in light of the central role of epithelial stem cells in tissue regeneration and tumor initiation. Stem cell behavior is strongly influenced by the cellular microenvironment, and intrinsic changes in proliferative or self-renewal potential may remain phenotypically silent in intact tissues while becoming apparent under conditions of stress or oncogenic signaling [1–4,32–35]. Moreover, oncogenic mutations do not act in isolation; their biological consequences are strongly influenced by the differentiation state and transcriptional identity of the cell in which they arise [32–35].

KRAS is a central regulator of cellular proliferation and differentiation through the RAF–MEK–ERK/MAPK signaling cascade, and activating KRAS mutations are important drivers of epithelial tumorigenesis [36–40]. However, oncogenic KRAS does not produce identical outcomes in all epithelial contexts, indicating that the cellular state of the target cell is a critical determinant of oncogenic response [36–40]. The possibility that an altered p63 isoform could modify the intrinsic state of epithelial stem cells and thereby influence their response to oncogenic KRAS provides a potential mechanistic link between p63 isoform biology and squamous carcinogenesis.

Here, we investigated the biological consequences of p63 exon 4 deficiency using a genetically defined mouse model and primary epithelial stem cell cultures. We found that heterozygosity for the p63 exon 4 deletion markedly enhanced epithelial dysplasia in mice expressing oncogenic Kras^G12D^, revealing a genetic interaction between altered p63 function and oncogenic KRAS signaling. DNA binding analysis suggests that exon 4 does not simply determine the ability of p63 to bind chromatin but contributes to the specificity of p63-associated transcriptional regulation. These findings support a model in which Δ4-p63 establishes a distinct epithelial stem cell state characterized by enhanced clonogenic potential and altered p63-associated chromatin regulation. Although this state remains largely compensated under normal physiological conditions, it becomes functionally apparent under oncogenic stress. The association of *Δ4-p63* expression with human SCC, together with its effects on epithelial stem cell properties and its cooperation with oncogenic KRAS, suggests that isoform-specific remodeling of p63 function may represent an underappreciated mechanism linking epithelial stem cell biology to squamous carcinogenesis.

## Results

### Generation and Characterization of Mice Carrying a p63 Exon 4-Deficient Allele

To investigate the biological function of the p63 exon 4 region, we generated a conditional mouse allele in which exon 4 of the *p63* gene was flanked by loxP sites (p63^flox-E4^) (Figure 1A). The targeting vector was introduced into mouse FLP Hybrid (129/SvEv × C57BL/6) embryonic stem (ES) cells by electroporation. During expansion of the targeted ES clones, the neomycin-resistance cassette flanked by FRT sites was removed by FLP recombination. Targeted ES clones were identified and verified by Southern blot analysis of genomic DNA (Figure 1B). Two independently verified ES clones were used for blastocyst injection, and germline transmission was established to generate heterozygous p63^+/flox-E4^ mice.

To determine whether the presence of the loxP sites and surrounding sequences affected p63 function, homozygous p63^flox-E4/flox-E4^ mice were examined. These mice developed without gross abnormalities and were viable into adulthood, indicating that the unrecombined floxed allele did not overtly disrupt p63 function.

Deletion of exon 4 results in an in-frame truncation of p63 rather than a frameshift (Supplementary Figure S1). We therefore designated the resulting protein as Δ4-p63. Because generation of an antibody specifically recognizing the exon 3–exon 5 junction was unsuccessful, we instead exploited the differential recognition of two antibodies directed against distinct p63 epitopes (Figure 2A). The mouse monoclonal antibody 4A4 recognizes an epitope that is absent from Δ4-p63, whereas the rabbit monoclonal antibody Cα recognizes the C-terminal region shared by WT p63 and Δ4-p63. In transiently transfected H1299 cells, 4A4 detected WT p63 but not Δ4-p63, whereas Cα detected both proteins by Western blotting (Figure 2B). Thus, WT p63 and Δ4-p63 could be distinguished as 4A4^+^/Cα^+^ and 4A4^−^/Cα^+^, respectively.

We next asked whether Δ4-p63-expressing cells could be detected *in vivo* in the absence of WT p63. Using K14-Cre to delete the floxed exon 4 allele in the epidermis, we examined skin sections from K14-Cre;p63^+/flox-E4^ and K14-Cre;p63^flox-E4/flox-E4^ embryos at embryonic day 12 (E12). K14-Cre-mediated recombination occurs in the epidermis during early embryonic development [41]. In K14-Cre;p63^flox-E4/flox-E4^ embryos, Cα staining was maintained throughout the basal layer, whereas 4A4 staining was absent from a subset of basal cells (Figure 2C). In contrast, basal cells in K14-Cre;p63^+/flox-E4^ embryos remained positive for both 4A4 and Cα. These findings demonstrate that the 4A4^−^/Cα^+^ population corresponds to cells expressing Δ4-p63 and establish the ability to detect, although indirect, the mutant isoform *in vivo*.

### Heterozygous p63 Exon 4 Deficiency Does Not Cause Spontaneous Tumors

Because *Δ4-p63* expression is associated with human SCC [30], we next asked whether expression of Δ4-p63 was sufficient to induce spontaneous tumor development in mice. WT and p63 exon 4 heterozygous mice were monitored for 76 weeks (WT, n = 95; p63^+/flox-E4^, n = 161). During the observation period, 97% of WT mice and 88% of p63 exon 4 heterozygous mice remained free of spontaneous tumors, as determined by disease-free survival analysis (Figure 3A,B). Thus, heterozygous expression of Δ4-p63 was not sufficient to induce spontaneous epithelial tumor formation.

A subset of p63 exon 4 heterozygous mice (12%) developed mild eyelid inflammation and cataracts, which were associated with ocular irritation and were likely secondary to corneal scratching (Figure 3C). No evidence of spontaneous tumors was detected in epithelial tissues examined during the study. These findings indicate that despite the association of Δ4-p63 with human SCC, its expression in the heterozygous mouse background is insufficient by itself to induce spontaneous tumor formation.

### p63 Exon 4 Deficiency Enhances Oncogenic KRAS-Induced Epithelial Dysplasia

The absence of spontaneous tumors in Δ4-p63-expressing mice suggested that additional oncogenic signals might be required to reveal the biological consequences of exon 4 deficiency. Oncogenic KRAS is a potent driver of epithelial hyperproliferation through constitutive activation of downstream signaling pathways, including the RAF–MEK–ERK and PI3K–AKT pathways [36,37]. In particular, oncogenic Kras^G12D^ produces pronounced hyperproliferation of oral epithelial cells and can lead to papilloma formation, demonstrating the marked sensitivity of oral squamous epithelium to oncogenic KRAS signaling [42].

To determine whether Δ4-p63 modifies the response to oncogenic KRAS, we generated mice carrying both the p63 exon 4 heterozygous allele and oncogenic Kras^G12D^ under the control of a tamoxifen-inducible K14-CreERT transgene (K14-CreERT;Kras^+/G12D^;p63^+/flox-E4^, n = 13). As controls, we generated K14-CreERT;p63^+/flox-E4^ mice (n = 13), K14-CreERT;Kras^+/G12D^ mice (n = 14), and K14-CreERT mice (n = 14).

Following tamoxifen administration once daily for five consecutive days, mice were monitored for 3–8 weeks. K14-CreERT;Kras^+/G12D^ mice and K14-CreERT;Kras^+/G12D^;p63^+/flox-E4^ mice developed severe epithelial dysplasia involving the lips, tongue, and oral mucosa, resulting in marked enlargement of the oral tissues and impaired food and water intake (Figure 4A,B). The oral masses and lip enlargement were more pronounced in the double-mutant mice than in mice expressing oncogenic Kras^G12D^ alone. In contrast, neither K14-CreERT nor K14-CreERT;p63^+/flox-E4^ mice developed obvious oral epithelial dysplasia during the observation period (Figure 4B).

Histological analysis confirmed the enhanced epithelial phenotype in the double-mutant animals. Consistent with previous reports [42], expression of Kras^G12D^ produced marked basal cell hyperplasia in oral epithelia, including the lip (Figure 5A,C). This basal hyperplasia was significantly greater in K14-CreERT;Kras^+/G12D^;p63^+/flox-E4^ mice than in K14-CreERT;Kras^+/G12D^ mice (Figure 5A,C). In contrast, oral epithelial basal layers of K14-CreERT;p63^+/flox-E4^ mice were comparable to those of K14-CreERT controls. Thus, p63 exon 4 deficiency alone did not show significant hyperplasia of the basal layers but enhances oncogenic Kras^G12D^-induced epithelial hyperplasticity.

### p63 Exon 4 Deficiency Increases the Clonogenic Potential of Epithelial Stem Cells

Because p63 is required for the proliferative and clonogenic potential of stem cells in stratified epithelia [11,12], we next examined whether exon 4 deficiency altered the intrinsic proliferative capacity of epithelial stem cells. Primary epidermal keratinocytes were isolated from WT and p63 exon 4 heterozygous littermates and evaluated using the clonogenic culture system originally developed by Green and colleagues [13]. In this assay, epithelial stem cells generate holoclones with high proliferative and long-term self-renewal capacity, whereas meroclones and paraclones represent progressively more differentiated populations with reduced proliferative potential [13,14].

Primary keratinocytes isolated from newborn or one-month-old mice were seeded at low density onto mitotically inactivated 3T3-J2 feeder cells. After two weeks, colony formation was assessed, and a subset of cultures was serially passaged for additional analysis (Figure 6A). WT keratinocytes formed compact colonies with relatively limited expansion, whereas keratinocytes derived from p63 exon 4 heterozygous mice consistently generated substantially larger colonies (Figure 6B). The colonies derived from exon 4-deficient mice retained the overall morphology of holoclone-like colonies but expanded more extensively than their WT counterparts.

This difference was not restricted to early culture. Following serial passage, p63 exon 4-deficient keratinocytes continued to form larger colonies than WT keratinocytes (Figure 6B). The persistence of this phenotype during prolonged culture argues against transient colony enlargement caused solely by progression toward a more differentiated meroclone state. Rather, the results indicate that epithelial cells carrying the p63 exon 4-deficient allele possess an intrinsically enhanced clonogenic capacity.

Importantly, these findings provide a cellular phenotype that was not evident from histological examination of intact skin. Thus, although p63 exon 4 heterozygous mice did not display an overt epithelial abnormality *in vivo*, their epithelial stem/progenitor cells exhibited a reproducible increase in clonogenic growth when removed from the tissue environment.

### WT p63 and the p63/Δ4-p63 Complex Exhibit Similar Global DNA-Binding Profiles but Distinct Preferential Regulatory Associations

To investigate the molecular basis of the altered clonogenic phenotype, we next compared the genomic DNA-binding profiles of p63 and p63/Δ4-p63 in primary keratinocytes derived from WT and p63 exon 4 heterozygous mice, respectively, using chromatin immunoprecipitation followed by high-throughput sequencing (ChIP-seq). It has been shown that p63 binds DNA as tetramers with high affinity [43]. As all p53 family members bind DNA as tetramers irrespective of sequence diversity during evolution or the mutation [43], we assume that Δ4-p63 forms tetramers with WT p63 or alone in heterozygous mice. Because Δ4-p63 retains the C-terminal epitope recognized by the Cα antibody whereas the 4A4 epitope is absent, the Cα antibody was used to immunoprecipitate both p63 and p63/Δ4-p63.

Genome-wide analysis revealed that p63 and p63/Δ4-p63 exhibited highly similar overall DNA-binding profiles, including substantial overlap at canonical p63-responsive regulatory regions (Figure 7A). Thus, deletion of exon 4 did not abolish the ability of p63 to associate with chromatin.

Despite this overall similarity, closer examination identified distinct regulatory regions preferentially associated with p63 or p63/Δ4-p63 (Figure 7B). WT p63 showed preferential association with regulatory regions linked to Gata2, Gata6, Gata1, Gata4, Foxk1, Foxo3, Gata3, Trps1, and Elf5. In contrast, p63/Δ4-p63 showed preferential association with regulatory regions linked to Npas, Arnt, Bmal1, Klf14, Elk4, Npas2, and Ar.

These findings demonstrate that exon 4 deletion does not simply eliminate p63 DNA binding. Instead, p63 and p63/Δ4-p63 retain broadly similar genomic occupancy while displaying differences in preferential association with specific regulatory regions. The ChIP-seq data therefore provide a molecular correlate for the altered epithelial stem cell phenotype observed in the clonogenic assay and identify a distinct set of transcriptional regulatory loci associated with Δ4-p63.

## Discussion

The present study identifies exon 4 of p63 as an important determinant of epithelial stem cell behavior and provides evidence that Δ4-p63, an aberrantly spliced p63 isoform, can influence squamous epithelial carcinogenesis without producing an overt phenotype in normal tissue. Our findings indicate that Δ4-p63 should not simply be regarded as a hypomorphic or loss-of-function form of p63. Rather, Δ4-p63 retains substantial chromatin-binding activity while exhibiting preferential association with a distinct subset of regulatory regions, is associated with enhanced epithelial stem cell clonogenicity, and cooperates with oncogenic KRAS to promote epithelial dysplasia. These findings support a model in which Δ4-p63 establishes a latent or “primed” epithelial state that is relatively compensated under normal physiological conditions but becomes apparent when epithelial cells encounter proliferative or oncogenic stress.

### p63 is a central regulator of epithelial development and stem cell potential

The essential role of p63 in stratified epithelial biology has been established by genetic studies demonstrating that p63-deficient mice exhibit profound defects in limb, craniofacial, epidermal, and other ectoderm-derived structures [9,10]. p63 expression is particularly prominent in basal and progenitor populations of stratified epithelia, suggesting an important role in maintaining cells with proliferative potential [11]. Subsequent studies demonstrated that p63 is required not only for epithelial development but also for the maintenance of proliferative potential in mature stratified epithelia [12,21]. Conditional depletion or knockdown of p63 in keratinocytes results in cell cycle arrest and impaired epithelial proliferation, while p63 is strongly expressed in highly clonogenic epithelial cells [12,21].

The clonogenic assay developed through the work of Green and colleagues provided an important functional framework for understanding epithelial stem cell behavior. Keratinocyte colonies can be classified according to their proliferative potential into holoclones, meroclones, and paraclones, with holoclones representing cells with the greatest long-term proliferative and self-renewal capacity [13,44]. The strong association between p63 expression and clonogenic potential subsequently provided evidence that p63 is functionally linked to the epithelial stem cell compartment [12]. Importantly, p63 is now recognized as a transcriptional regulator that coordinates multiple aspects of epithelial stemness, proliferation, differentiation, and tissue regeneration [24].

Our results extend these observations in an unexpected direction. Whereas complete loss of p63 severely compromises epithelial stem cell proliferation, heterozygous deletion of exon 4 resulted in an increase in clonogenic potential. Thus, p63 exon 4 does not simply contribute to the overall quantity of p63 activity; rather, it appears to influence the qualitative properties of p63-dependent epithelial stem cell regulation.

### Enhanced clonogenicity reveals a phenotype that is masked in intact epithelium

One of the most informative observations in this study is the discrepancy between the apparently normal skin of p63 exon 4 heterozygous mice and the markedly enhanced clonogenicity of epithelial cells isolated from the same animals. Histological examination did not reveal obvious differences between WT and heterozygous skin, irrespective of age. In contrast, primary epithelial cultures derived from heterozygous animals consistently produced larger colonies and exhibited greater clonogenic potential. This distinction is important because epithelial stem cell behavior *in vivo* is strongly influenced by the surrounding tissue environment. Stem cells are regulated by interactions with neighboring cells, the extracellular matrix, growth factors, inflammatory signals, and systemic factors [45]. Consequently, a change in intrinsic stem cell potential does not necessarily produce an obvious phenotype at the tissue level when compensatory mechanisms remain intact.

The persistence of the phenotype during serial passage is particularly significant. Transiently enlarged colonies can occur during the transition between clonogenic states, and the distinction between holoclones and meroclones is based in part on their long-term proliferative behavior and colony morphology [13,47]. Cells undergoing progressive differentiation may temporarily generate relatively large colonies but ultimately lose extensive proliferative capacity. In contrast, the continued presence of larger colonies in Δ4-p63-derived cultures after repeated passage suggests that the phenotype is not simply a consequence of transient differentiation-associated proliferation. Rather, exon 4 deficiency appears to alter an intrinsic property of the epithelial stem cell population.

This finding may also explain why the phenotype is not readily apparent in intact skin; however, the altered proliferative potential becomes readily evident in culture. The physiological epithelial niche may compensate for the increased intrinsic proliferative capacity of Δ4-p63-associated stem cells. Once cells are removed from the tissue and subjected to the selective conditions of clonogenic assays, this intrinsic difference may become more apparent. This distinction between tissue homeostasis and intrinsic stem cell potential may be particularly relevant to tumor initiation, because cellular properties that remain silent under normal conditions can become advantageous when oncogenic signaling or tissue stress is introduced.

### Exon 4 deficiency does not simply abolish p63 function

The ChIP-seq results provide an important mechanistic clue to the unusual phenotype produced by exon 4 deletion. p63 and p63/Δ4-p63 exhibited highly similar genome-wide DNA-binding patterns. This observation indicates that deletion of exon 4 does not globally eliminate the ability of p63 to associate with chromatin.

p63 functions through extensive interactions with cis-regulatory elements and chromatin-associated factors. Genome-wide studies have demonstrated that p63 occupies large numbers of enhancer and promoter regions in epithelial cells and contributes to the establishment and maintenance of epithelial-specific transcriptional programs [24]. p63 can also cooperate with other transcription factors, including KLF14, to establish epithelial enhancer landscapes [24]. Therefore, the biological activity of p63 cannot be understood solely from its ability to bind DNA; the identity of the regulatory regions occupied and the transcriptional cofactors recruited are also likely to be important determinants of its function.

In this context, the similar global binding profiles of p63 and p63/Δ4-p63, combined with their distinct preferential binding to subsets of regulatory regions, are particularly informative. WT p63 preferentially associated with regulatory regions linked to Gata2, Gata6, Gata1, Gata4, Foxk1, Foxo3, Gata3, Trps1, and Elf5. In contrast, p63/Δ4-p63 preferentially associated with regulatory regions linked to Npas, Arnt, Bmal1, Klf14, Elk4, Npas2, and Ar.

Although ChIP-seq occupancy alone cannot establish whether a bound regulatory element is transcriptionally activated or repressed, the differential distribution of p63 occupancy suggests that exon 4 contributes to transcriptional specificity. Thus, Δ4-p63 may retain the general chromatin-binding properties of p63 while changing the subset of regulatory elements preferentially recognized or stabilized by the protein.

This distinction is important because it changes the conceptual interpretation of exon 4 deficiency. A classical loss-of-function allele would be expected primarily to reduce p63-dependent transcription. Our findings instead suggest a qualitative change in p63 function. Δ4-p63 may therefore be better considered a functionally distinct p63 isoform that reshapes the p63-associated transcriptional landscape rather than simply reducing total p63 activity.

### p63 isoform diversity may provide a mechanism for changing epithelial cell states

The *p63* gene produces multiple isoforms through alternative promoter usage and alternative splicing, and these isoforms can have distinct and sometimes opposing biological activities [16]. ΔNp63 is particularly abundant in basal epithelial cells and has been extensively studied as a regulator of epithelial proliferation, differentiation, and lineage identity. The functional diversity of p63 isoforms raises the possibility that changes in isoform composition can alter epithelial cell behavior even when total p63 expression remains relatively unchanged.

Our previous work provided an early indication that aberrant expression of a p63-family isoform may be associated with squamous carcinogenesis. We reported transcriptional dysregulation of the *p73L* (*TP63)* locus in human SCC and identified ΔNp73L (equivalent to Δ4-p63) as a previously unrecognized isoform that was expressed in squamous carcinomas but not detected in normal skin [30]. The same study identified loss of expression of p51 (or TAp63), a potential tumor suppressor, and suggested that ΔNp73L could interfere with p53/p51-family transcriptional activity [30]. These observations raised the possibility that qualitative alterations in p63-family isoform expression may contribute to SCC development.

The present findings extend that concept by demonstrating that an altered p63 isoform can affect epithelial stem cell behavior *in vivo* and *ex vivo*. The emergence or increased representation of an otherwise minor p63 isoform may therefore have consequences that cannot be predicted simply from measurements of total p63 expression.

Squamous epithelial carcinogenesis is particularly dependent on interactions between oncogenic signaling and the transcriptional programs that maintain epithelial identity [27,28]. Our finding that Kras^G12D^ produces substantially more severe epithelial dysplasia in the p63 exon 4 heterozygous background suggests that the altered p63 state may modify the response of epithelial cells to oncogenic RAS signaling.

Importantly, however, our data do not establish that Δ4-p63 directly activates the KRAS/MAPK pathway. The genetic interaction observed *in vivo* is compatible with several mechanisms. Δ4-p63 could increase the number or persistence of epithelial cells capable of responding to oncogenic KRAS, alter the transcriptional response to downstream MAPK signaling, or change the threshold at which oncogenic signaling produces abnormal epithelial expansion. The preferential association of p63/Δ4-p63 with regulatory regions linked to factors such as ELK4 and KIF14 is intriguing in this regard, but whether these factors directly mediate the interaction between Δ4-p63 and KRAS remains to be determined.

Thus, we favor a model in which Δ4-p63 establishes a permissive epithelial state rather than one in which Δ4-p63 functions as a direct activator of KRAS signaling.

### A potential link between epithelial stem cell state and squamous carcinogenesis

The combination of the clonogenic and Kras^G12D^ phenotypes suggests an important conceptual model. Δ4-p63-associated epithelial cells possess increased clonogenic potential but do not spontaneously develop prominent dysplasia under normal conditions. The acquisition of oncogenic KRAS, however, dramatically exposes this altered cellular state.

This observation is consistent with the broader concept that cancer susceptibility is influenced not only by the presence of oncogenic mutations but also by the cellular state in which those mutations arise [32–35]. Stem and progenitor cells with enhanced self-renewal capacity may provide a particularly favorable substrate for oncogenic transformation because they possess the ability to persist, expand, and generate differentiated progeny. Alterations in the transcriptional state of such cells could therefore influence the consequences of otherwise similar oncogenic mutations.

Our findings suggest that Δ4-p63 may function in this manner. Rather than directly inducing dysplasia, Δ4-p63 may increase the intrinsic capacity of epithelial stem cells to expand and thereby create a population that is more responsive to oncogenic stimuli. Oncogenic KRAS may then convert this permissive state into overt epithelial dysplasia.

This model also provides a potential explanation for why the Δ4-p63 phenotype is subtle in normal skin. The intact epithelium may remain morphologically normal because tissue-level regulatory mechanisms constrain the increased proliferative potential. The same cells, however, may respond differently when exposed to oncogenic signaling or when removed from their normal niche.

### Implications for squamous carcinogenesis and human SCC

The association between aberrant p63-family isoform expression and human SCC observed in our earlier work [30], together with the present genetic and functional findings, raises the possibility that p63 isoform switching represents an underappreciated mechanism in squamous carcinogenesis.

The conventional view of p63 in SCC has focused largely on the high expression of ΔNp63 and its ability to support squamous lineage identity, proliferation, and tumor maintenance [25–29]. Our findings suggest an additional layer of complexity: the biological consequences of p63 expression may depend not only on its overall abundance but also on the precise isoform composition of the p63 pool.

This distinction could be particularly relevant in tumors because cancer cells frequently exploit alternative transcriptional states rather than simply increasing the activity of a single pathway. An isoform such as Δ4-p63, which retains substantial chromatin-binding activity but has altered regulatory preferences, could therefore provide a mechanism for remodeling an existing epithelial transcriptional network without completely disrupting epithelial identity.

The fact that Δ4-p63 is associated with human SCC is consistent with this possibility. However, the present study does not establish whether Δ4-p63 expression is an initiating event in human SCC, a consequence of transformation, or a state selected during tumor evolution. Determining the temporal relationship between Δ4-p63 emergence and oncogenic transformation will require analysis of appropriate human premalignant lesions and additional experimental models.

### Limitations and future directions

Several limitations should be considered. First, the ChIP-seq experiments identify differential p63 occupancy but do not directly demonstrate changes in transcription. RNA-seq, chromatin accessibility analysis, and functional interrogation of candidate target genes would be required to determine which Δ4-p63-associated regulatory regions are functionally important.

Second, although the clonogenic phenotype strongly indicates altered epithelial stem cell potential, the precise cellular mechanism remains unresolved. Increased self-renewal, altered cell cycle regulation, delayed differentiation, or changes in survival could each contribute to the observed phenotype. The persistence of the phenotype through serial passage argues against transient differentiation-associated expansion, but it does not distinguish among these mechanisms.

Third, the genetic interaction between Δ4-p63 and Kras^G12D^ establishes cooperation but does not identify the molecular pathway connecting the two factors. In particular, our present data do not demonstrate direct activation of the RAF–MEK–ERK pathway by Δ4-p63. Future studies would be required to determine whether Δ4-p63 modifies MAPK signaling, alters transcriptional responses downstream of MAPK, or instead changes the cellular substrate upon which oncogenic KRAS acts.

Finally, the present study focuses on heterozygous p63 exon 4 deficiency. Because homozygous deletion is neonatal lethal, the biological consequences of complete Δ4-p63 expression or complete loss of exon 4-containing isoforms cannot be assessed using this model. Nevertheless, the heterozygous state may be particularly relevant to tumorigenesis because partial changes in transcription factor activity can produce cellular phenotypes without causing catastrophic developmental abnormalities.

## Conclusions

In summary, our findings identify p63 exon 4 as an important determinant of epithelial stem cell behavior and p63-dependent transcriptional specificity. Heterozygous exon 4 deficiency produces no obvious abnormality in intact skin but results in a persistent increase in epithelial stem cell clonogenic potential. At the molecular level, Δ4-p63 retains broad chromatin-binding activity while exhibiting preferential association with a distinct set of regulatory regions. In the presence of oncogenic Kras^G12D^, this altered cellular state is associated with markedly enhanced epithelial dysplasia.

Together with our previous observation of aberrant p63-family isoform expression in human SCC [30], these findings support a model in which Δ4-p63 represents more than a defective form of p63. Instead, Δ4-p63 may establish a distinct epithelial stem cell state that is compatible with normal tissue homeostasis but becomes permissive for oncogenic transformation. Isoform-specific remodeling of p63 function may therefore represent an important mechanism linking epithelial stem cell biology, oncogenic signaling, and squamous carcinogenesis.

## Methods

### Deletion of Exon 4 of the *p63* Gene by Homologous Recombination

Gene targeting was performed at the Ingenious Targeting Laboratory (Ronkonkoma, NY). The targeting vector was comprised of the 5’ homologous arm, the exon 4-containing fragment followed by the PGK-Neo^r^ cassette, and the 3’ homologous arm. Two loxP sites (closed ovals) and two flp sites (closed circles) were inserted as shown in Figure 1A. The entire targeting vector was verified by direct sequencing. Gene targeting was performed in FRT-hybrid (129/SvEv × C57BL/6) embryonic stem cells (ESCs), thus allowing the deletion of the PGK-Neo^r^ cassette during the expansion of the targeted ESC clones. Screening of the targeted ESC clones was carried out by PCR with the primer pair GT (5’-gtc cgt gtc gcg aag ttc cta tac ttt c-3’) and A3 (5’-tcc ttg ggc atg gct tat ctt gtc-3’), followed by verification by Southern blot analysis. The Southern blot probe, located outside the 3’ homologous arm (see Figure 1A), detected the 8,831 bp DNA fragment from WT allele and the 6,233 bp DNA fragment from the targeted allele upon digestion with the restrict enzyme MfeI. Two correctly targeted ESC clones were microinjected into C57BL/6 blastocysts to obtain chimeric mice. Male chimeras were then mated to female C57BL/6 mice to achieve germline transmission of the targeted allele (p63^flox-E4^ allele). The p63^flox-E4^ allele was fully sequenced to verify the correct gene targeting. Subsequent crossing of the p63^+/flox-E4^ mice with tissue-specific Cre-expressing mice (Jackson Lab, Bar Harbor, ME) deleted exon 4 of the *p63* gene (p63^Δ4^ allele) in a tissue-specific manner.

### Animals

C57BL/6, Actin-Cre, K14-Cre, K14-CreERT, and Kras^G12D^ mice were all purchased from the Jackson Laboratory (Bar Harbor, ME). p63^flox-E4^ mice and p63^Δ4^ mice were created in this study. All procedures were performed in accordance with a protocol approved by the Institutional Animal Care and Use Committee at the Boston University Medical Campus (protocol number: AN-15050). For staged embryos, the day of the vaginal plug was designated E0.5. When euthanizing adult mice, a two-step method designed to ensure a humane and irreversible death in compliance with American Veterinary Medical Association guidelines was performed. First, animals were placed in an environment with an elevated concentration of Isoflurane, typically two to three times the standard surgical dose. Unconsciousness was confirmed by toe pinching, and cervical dislocation was performed as a secondary physical procedure.

### Antibodies

The primary antibodies used in this study were mouse anti-p63 (4A4, Santa Cruz Biotechnology; 1:250), rabbit anti-p63Cα (D2K8X, Cell Signaling Technology; 1:500), and mouse anti-tubulin α (12G10, Developmental Studies Hybridoma Bank, University of Iowa, Iowa City, IA, USA; 1:500). Secondary antibodies used for immunofluorescence were Alexa 594-goat anti-mouse IgG and Alexa 488-goat anti-rabbit IgG (Molecular Probes). For Western blot analysis, horseradish peroxidase (HRP)-conjugated goat anti-mouse IgG (KPL) and HRP-conjugated goat anti-rabbit IgG (Cell Signaling Technology) were used.

### Southern Blot

Genomic DNA was digested with the restriction enzyme MfeI, separated on a 0.8% agarose gel, and transferred to nylon membranes (Sigma-Aldrich). To confirm homologous recombination, a 694 bp genomic fragment generated by PCR and targeted against 3’ external region was radiolabeled with [^32^P]dCTP (GE Healthcare) and used as a probe. To label the DNA fragments, the Rediprime II DNA Labeling System (GE Healthcare) was used. Positive clones were further confirmed by direct sequencing of genomic DNA prepared from the targeted ESC clones (Figure 1B).

### Western Blot

The following plasmids were used: pcDNA3 harboring human ΔNp63alpha cDNA, pcDNA3 harboring human Δ4-p63 (alpha) cDNA, and the empty pcDNA3. Twenty-four hours after the transfection, the H1299 cells were lysed in a protein lysis buffer containing 50 mM Tris-HCl (pH 6.8), 5% 2-mercaptoethanol, 2% sodium dodecyl sulfate, 10% glycerol, 1 mM phenylmethanesulfonyl fluoride and 1 mM sodium orthovanadate. After boiling for 5 min, whole cell extracts were subjected to 8–12% SDS-PAGE followed by Western blot.

### Tissue Preparation and Immunofluorescence

Mouse tissues were collected and fixed overnight in 10% formalin (Fisher Scientific). Subsequently, the issues were embedded in paraffin, sectioned, and stained with hematoxylin & eosin (H&E). Paraffin-embedded sections were cut into 6 μm sections and antigen retrieval was performed by incubating the slides in 0.01 M citric acid buffer (pH 6.0) at 95°C for 20 min. The sections were then blocked with 10% fetal bovine serum (FBS) (Atlanta Biologicals) at room temperature for 30 min. Subsequently, the treated slides were incubated with primary antibodies overnight at 4°C. After three washes in PBS containing 0.1% Tween 20 (PBST), the slides were incubated with fluorescently labeled secondary antibodies for 1 h at room temperature followed by counterstaining with Hoechst 33342 (Molecular Probes) for 1 min and washing in PBST for 10 min.

### Chromatin Immunoprecipitation Sequencing

ChIP-seq was performed on keratinocytes derived from WT and heterozygous p63 exon 4 mouse littermates using the SimpleChIP Plus Sonication Chromatin kit (Cell Signaling). Briefly, 1–5×10^7^ cells per reaction were collected, cross-linked with 1% formaldehyde, and quenched with a final concentration of 125 mM glycine. After washing with ice-cold PBS, the pellet was resuspended in lysis buffer (750 μl per 1×10^7^ cells). Lysate was sonicated until 60% to 90% of the chromatin were fragmented to smaller than 1 kb. One hundred μl of each sample was diluted 10 times in ChIP dilution buffer. 10 μl of ChIP-grade protein G and 10 μl of anti-p63 Cα antibody were complexed and incubated with the lysate at 4°C overnight. The immune complexes were purified according to the manufacturer’s instructions and the ChIP-DNA was reverse cross-linked and purified for DNA library construction. Deep sequencing and data analysis were performed in the Core facility at the Tufts Medical Center (Boston, MA).

### Tamoxifen Treatment

To induce the expression of Δ4-p63 or Kras^G12D^ or a combination of both, a series of K14CreERT mice were treated with Tamoxifen (Sigma-Aldrich). Tamoxifen was dissolved in corn oil overnight at a concentration of 10 mg/ml by rocking in a warm room and administered at 1 mg/100 μl per intraperitoneal (IP) injection. Mice were aged between seven and twelve weeks at the first injection. All mice received five injections every 24 hours over five days; control mice were injected with the corn oil vehicle only.

### Cell Culture

Human lung carcinoma H1299 cells were grown in DMEM containing 10% FBS, 10 U/ml penicillin and 100 μg/ml streptomycin in a humidified chamber at 37°C with 5% CO_2_. Co-culture with the 3T3-J2 feeder cells was performed as described [13]. Briefly, epithelial cells were seeded at a clonal density on Mitomycin C-treated 3T3-J2 cells (gift from H. Green, Harvard Medical School) in complete FAD (cFAD) media and grown in a humidified chamber at 37 °C with 5% CO_2_. To perform serial co-culture with 3T3-J2 cells, epithelial cells were harvested from the initial 3T3-J2 co-cultures by trypsinization in 0.25% trypsin for 5–10 min and equal numbers of epithelial cells were serially cultured with Mitomycin C-treated fresh 3T3-J2 cells with two-week intervals. To visualize epithelial colonies, culture plates were fixed in 10% buffered formalin for 10 min and stained with 1% Rhodamine B (Sigma-Aldrich, St. Louis, MO).

## Supplementary Material

Supplementary Files

This is a list of supplementary files associated with this preprint. Click to download.

- FigureS1.tif
- FigureS2.tif

## Figures and Tables

**Figure 1.**
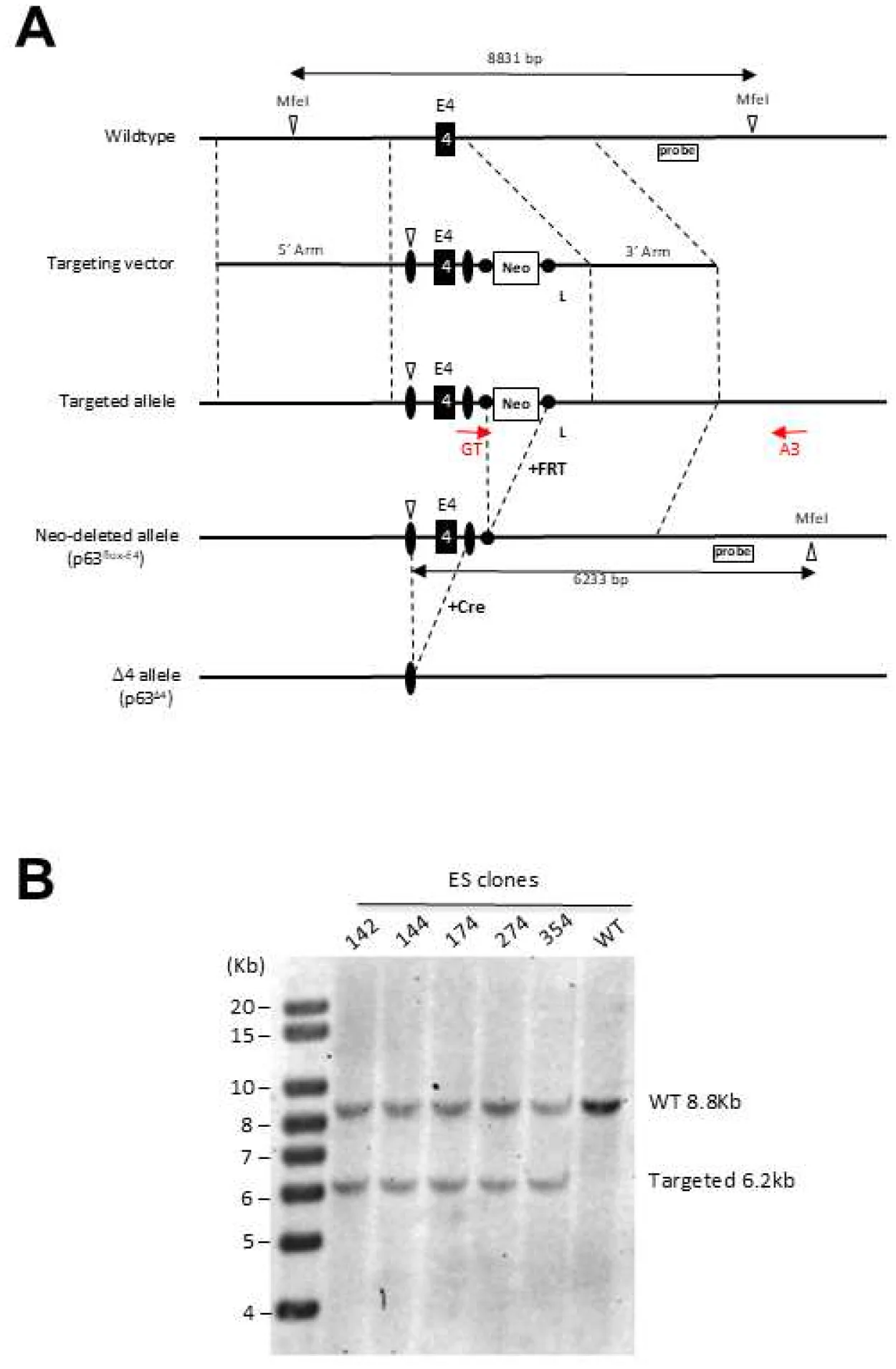
Deletion of exon 4 of the *p63* gene by gene targeting in mice. (A) Targeting strategy by homologous recombination. The DNA region around exon 4 of the *p63* gene is shown. The targeting vector included the 5 ’homologous arm, exon 4 followed by a neomycin cassette, and the 3 ’homologous arm. The ovals and circles represent the loxP and flp sequences, respectively. The positions of the PCR primers (GT and A3) and the probes for Southern blot (boxed) are shown. In Southern blot analysis, the correct homologous recombination reduces the size of the exon 4-containing MfeI fragment from 8,831 bp to 6,233 bp, as indicated. Removal of the neomycin cassette was carried out in ESCs during expansion. Subsequently, p63^+/flox-E4^ mice with Cre-expressing mice deleted exon 4, producing mice lacking exon 4-coding region of the *p63* gene. The deletion of exon 4 does not cause a frameshift, resulting in the generation of the intact p63 proteins lacking the exon 4-coding domain (see Supplemental Figure S1). (B) Southern blot analysis of DNA from the targeted ESC clones.

**Figure 2.**
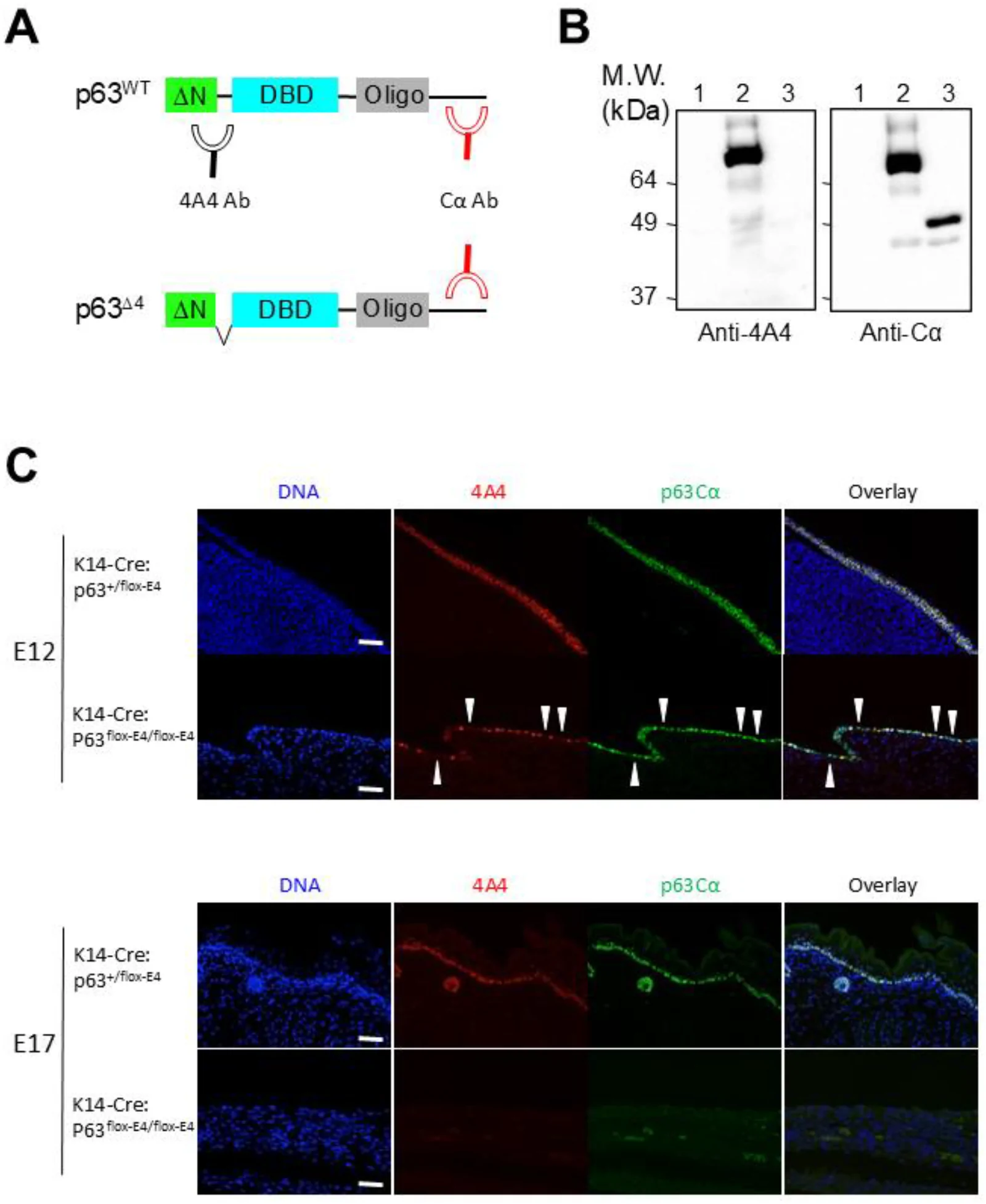
Detection of p63 by Western blot and immunofluorescence. (A) Recognition sites of wild-type (WT) p63 and Δ4-p63 proteins by the N-terminusspecific 4A4 and the C-terminus-specific anti-Cα antibodies. (B) Western blot analysis of the lung carcinoma H1299 cells transiently transfected with WT p63 (ΔNp63α) and Δ4-p63 (ΔNp63α lacking exon 4) plasmids, probed with 4A4 (left) and anti-Cα (right) antibodies. Mock transfectant with the empty pcDNA3 plasmid was used as a control (lane 1). (C) Immunofluorescence of developing epidermis of K14-Cre;p63^+/flox-E4^ and K14-Cre;p63^flox-E4/flox-E4^ mouse embryos stained with 4A4 (red), anti-Cα (green), and Hoechst 33342 (blue). Data shown are representative of embryonic day 12 (E12). Scale bars = 100 μm.

**Figure 3.**
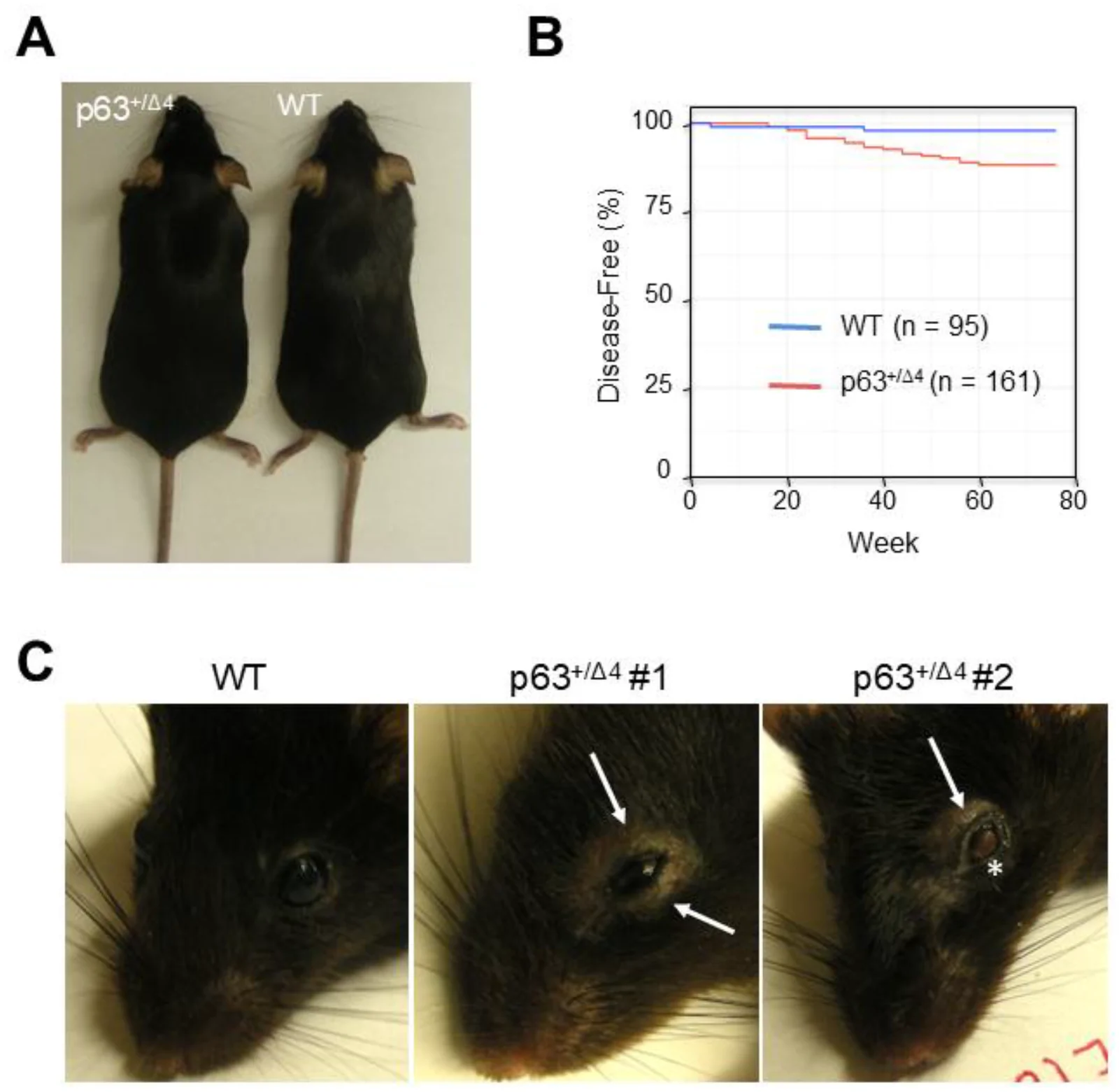
p63 exon4 mice do not develop cancers but develop inflammation of the eyelids and cataract. (A) Gross appearance of WT (right) and p63 exon 4 (left) mouse littermates at six months of age. (B) Disease-free curve of WT (blue) and p63 exon 4 (red) mice followed 76 weeks after birth. The loss of mutant mice was due to a mandatory euthanasia caused by inflammation of the eye region as determined by the attending veterinarians. (C) Inflammation in p63 exon 4 mice at the age of five months. Data shown are representative of animals with inflamed eyelids (arrows) and cataract (asterisk), respectively.

**Figure 4.**
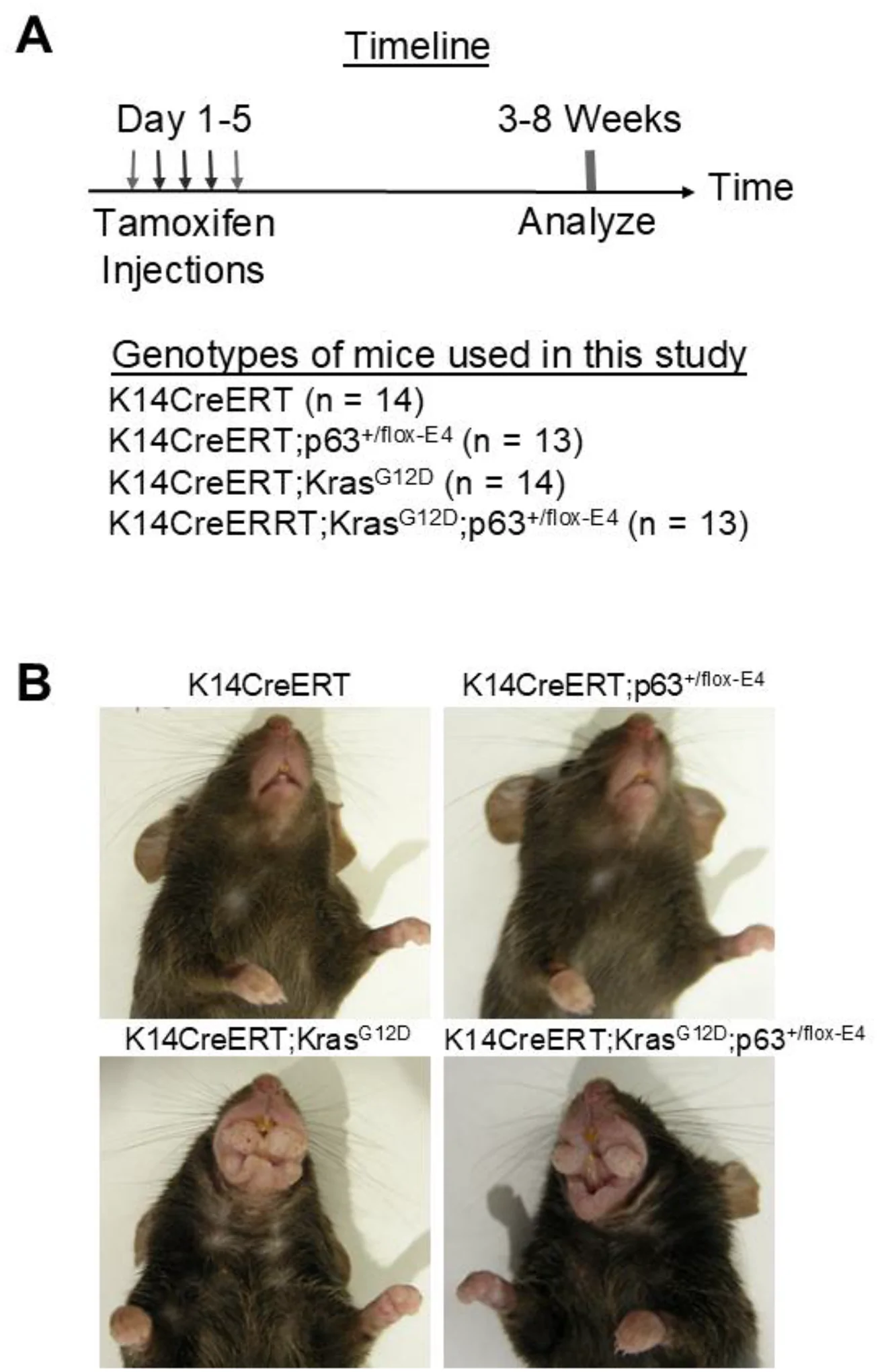
Δ4-p63 enhances Kras^G12D^-mediated epithelial transformation into dysplasia in mice. (A) Mice were given tamoxifen via IP every 24hrs for five consecutive days. Three to eight weeks after the last injection of tamoxifen, mice were euthanized and analyzed. Genotypes and number of animals used in this study are indicated. (B) Gross appearance of large masses developing in the lips and oral cavities in K14CreERT;Kras^+/G12D^ mice (lower left) and K14CreERT;Kras^+/G12D^; p63^+/flox-E4^ mice (lower right). Data shown are representative of each genotype with similar results.

**Figure 5.**
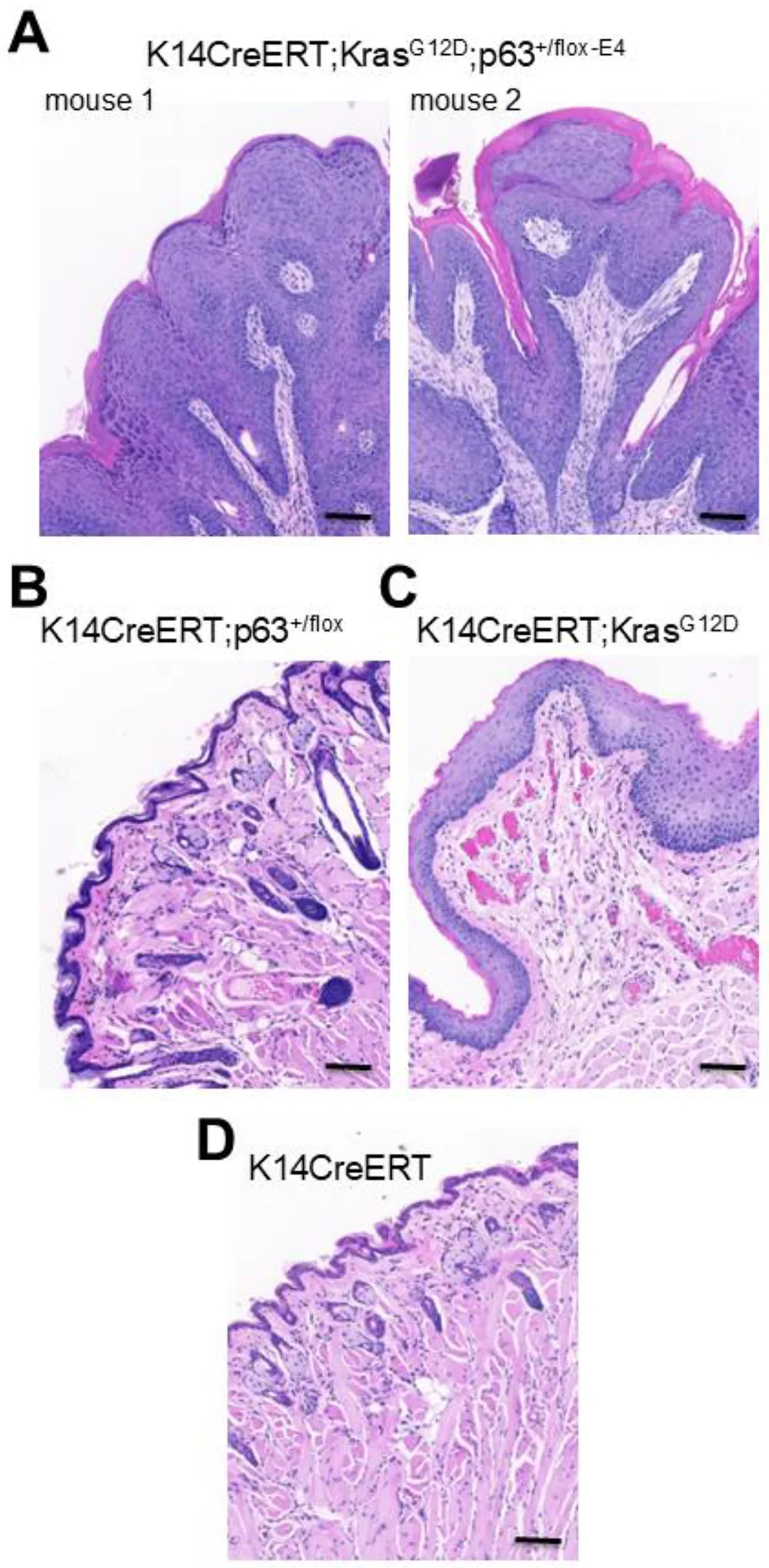
Histology of the lower lips in mice expressing Kras^G12D^, Δ4-p63, or the combination of both Kras^G12D^ and Δ4-p63. Data shown are continuous from Figure 4. (A) Representative images of two individual K14CreERT;Kras^+/G12D^;p63^+/flox-E4^ mice. (B-D) Data shown are representative of K14CreERT;p63^+/flox-E4^ mice (B), K14CreERT;Kras^G12D^ mice (C), and K14CreERT mice (D). Scale bars = 100 μm.

**Figure 6.**
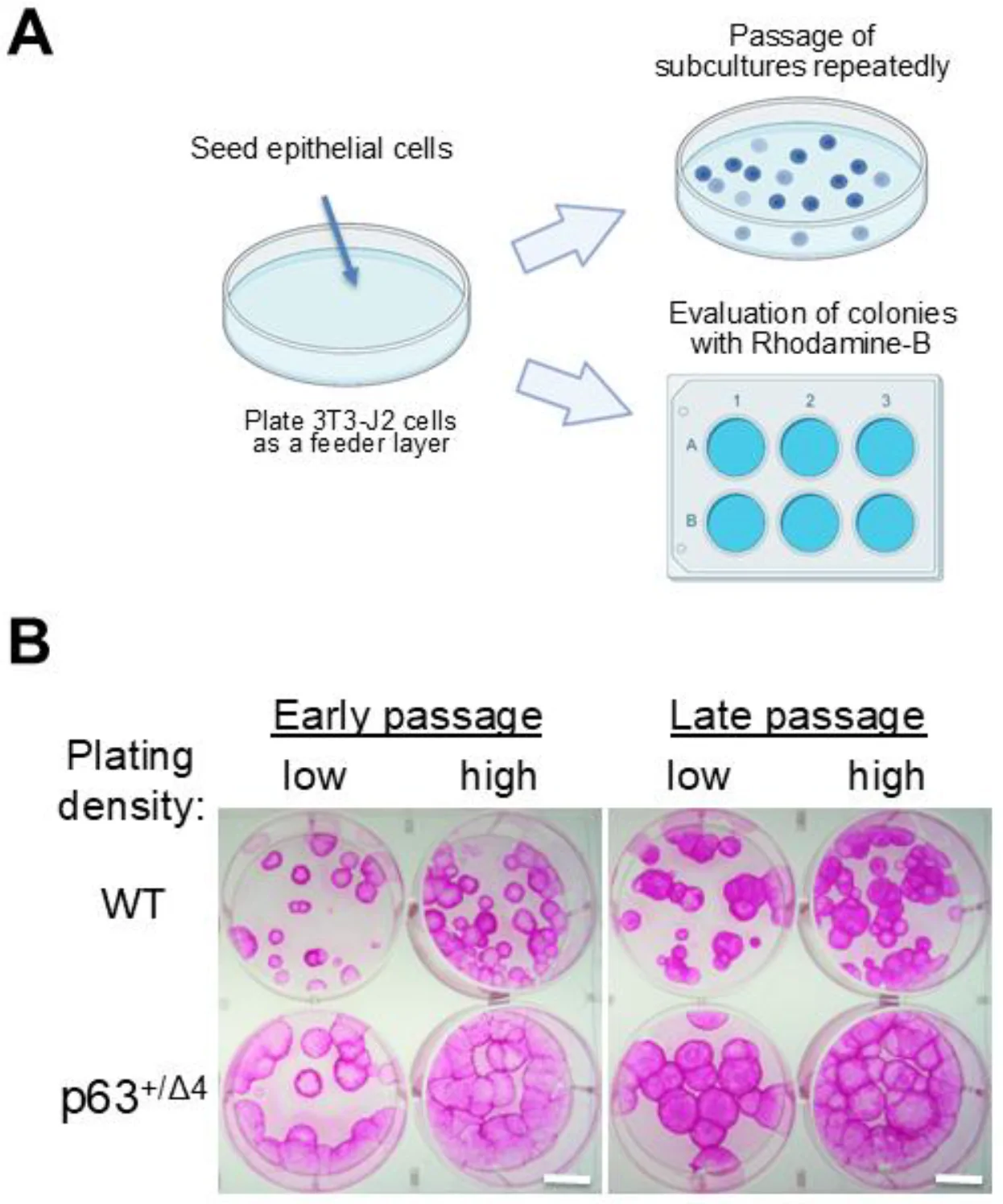
Increased proliferative capacity of epithelial stem cells in p63 exon 4 mice. (A) A schematic representation of the clonogenic culture of epithelial stem cells to assess their proliferative capacity. Epithelial cells were seeded on the mitotically arrested 3T3-J2 feeder layer in 10 cm plates (left). After two weeks of co-culture with 3T3-J2, the whole plates were trypsinized and passaged repeatedly to maintain the master plates (top right). In addition, in each passage, equal numbers of epithelial cells (low density: 100 cells, high density: 1,000 cells) were sub-cultured onto 3T3-J2 cells in 6-well plates to evaluate the colonies with Rhodamine-B staining (lower right). (B) Skin epithelial cells were prepared from newborn WT and p63 exon 4 mice and used in clonogenic analysis as depicted in (A). Early and late passages indicate the 2^nd^ and 10^th^ passages, respectively. Scale bars = 10mm.

**Figure 7.**
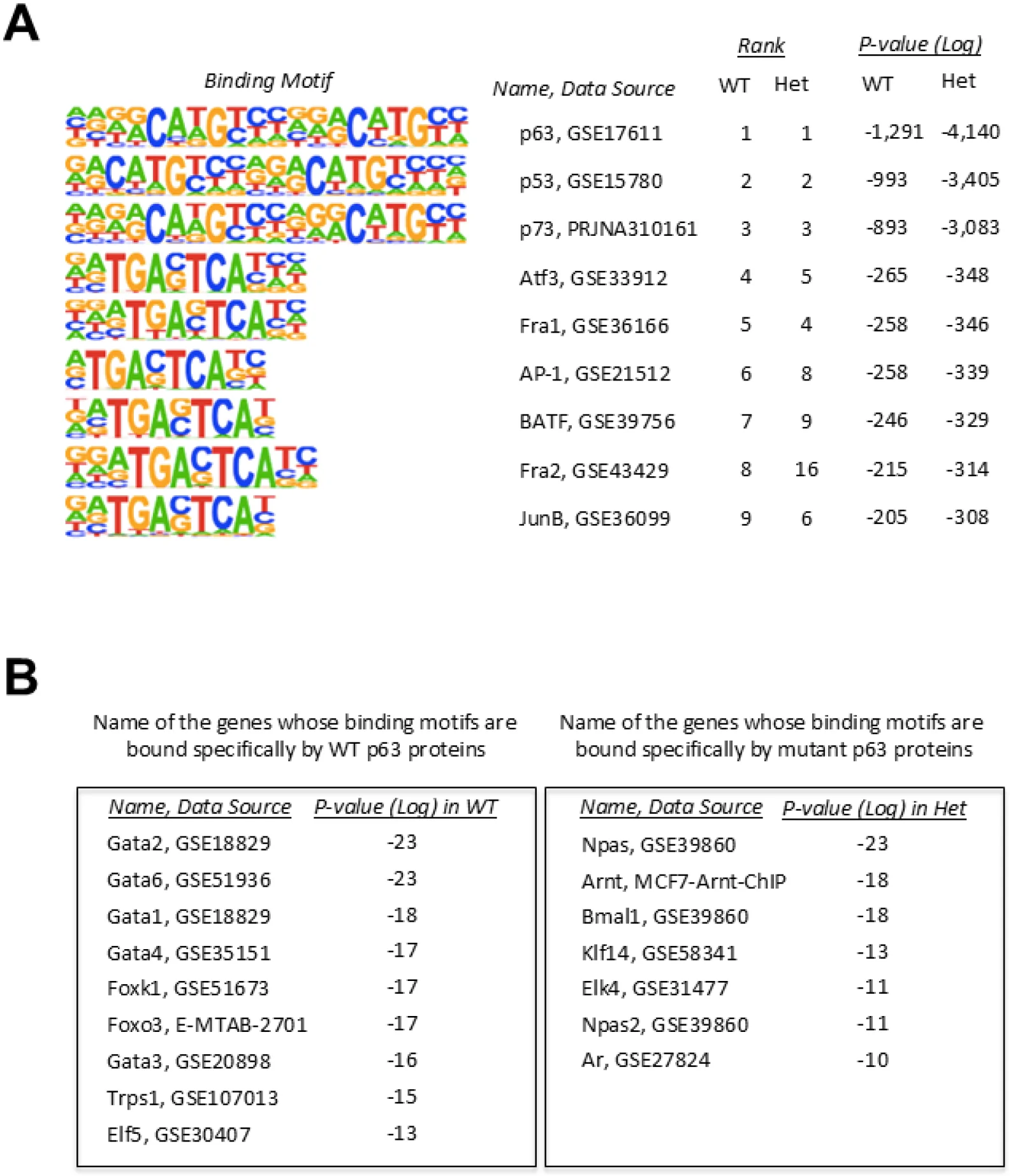
DNA binding activities of WT and mutant p63 proteins. Keratinocytes were prepared from the epidermis of WT and p63 exon 4 mouse embryos and the p63/DNA complexes were purified using anti-Cα antibodies, followed by DNA sequencing. (A) The list of the motifs with the greatest probabilities (the smallest *p*-value) for WT p63 (< −200 in *p*-value) was very similar to heterozygous p63 exon 4 protein complex. (B) The DNA binding motifs unique to WT (left) and mutant p63 (right) proteins are shown. The gene motifs with smaller *p*-value (Log) than −2 were included for comparison.

## Data Availability

All data generated or analyzed during this study are included in this published article. The ChIP-seq datasets generated during the current study are available in the NCBI-SRA repository under the Accession Number PRJNA1531960.
